# Vitamin A, D, and E Levels and Reference Ranges for Pregnant Women: A Cross-Sectional Study 2017–2019

**DOI:** 10.3389/fnut.2021.628902

**Published:** 2021-03-22

**Authors:** Fan Gao, Fanfan Guo, Yidan Zhang, Yufei Yuan, Dunjin Chen, Guiqin Bai

**Affiliations:** ^1^Clinical Research Center, The First Affiliated Hospital of Xi'an Jiaotong University, Xi'an, China; ^2^Department of Gynecology and Obstetrics, The First Affiliated Hospital of Xi'an Jiaotong University, Xi'an, China; ^3^Department of Gynecology and Obstetrics, The Third Affiliated Hospital of Guangzhou Medical University, Guangzhou, China

**Keywords:** vitamin A, pregnancy, vitamin D, vitamin E, reference range

## Abstract

**Background:** Pregnancy-specific vitamin reference ranges are currently not available for maternal vitamin management during pregnancy. This study aimed to propose pregnancy-specific vitamin reference ranges and to investigate the factors influencing vitamin levels during pregnancy.

**Methods:** A cross-sectional study that included pregnant women from 17 cities in 4 provinces in western China was conducted from 2017 to 2019. A total of 119,286 subjects were enrolled in the study. Serum vitamin A, vitamin D, and vitamin E levels were measured. A multivariable linear regression model and restricted cubic spline function were used to analyze the factors related to vitamin levels.

**Results:** The reference ranges for vitamin A, D, and E levels were 0.22–0.62 mg/L, 5–43 ng/mL, and 7.4–23.5 mg/L, respectively. A linear relationship was found between vitamin E level and age (β = 0.004; 95% confidence interval [CI], 0.0037–0.0042; *p* < 0.001), and a nonlinear relationship was found between vitamin D (*p* nonlinear = 0.033) and vitamin A levels and age (*p* nonlinear < 0.001). Season, gestational trimester, and regions were related to the levels of the three vitamins in the multivariable models (*p* < 0.05).

**Conclusions:** The lower limit of vitamin A during pregnancy was the same as the reference value currently used for the general population. The reference ranges of vitamins D and E during pregnancy were lower and higher, respectively, than the currently used criteria for the general population. Vitamin A, D, and E levels differed according to age, season, gestational trimester, and region.

## Introduction

Maternal vitamin levels during pregnancy are closely linked to the health and survival of mothers and their offspring (1–10). From 1995 to 2005, ~19 million pregnant women had vitamin A deficiency and ~9.8 million pregnant women had night blindness worldwide, mostly from Southeast Asia and Africa (11). A systematic review in 2016 reported that more than half of pregnant women and newborns had vitamin D deficiency in five global regions (12). The vitamin levels in pregnant women remains a global health concern, especially in developing countries.

Many health organizations have defined thresholds for all types of vitamin deficiency (13–15). However, the vitamin reference ranges during pregnancy are still not specified (16, 17). Owing to the physiological changes during pregnancy, the vitamin reference values may differ from those of healthy individuals (16). Recent studies that focused on the effects of multivitamin supplementation on maternal and fetal outcomes had inconsistent conclusions (4, 7, 9, 10, 18–21). The differences may be attributed to the different supplement programs, different regions, and lack of vitamin reference ranges. Vitamin A, D, and E levels are commonly studied to identify any relation with adverse perinatal outcomes (22–25). Especially for vitamin D, establishing prenatal requirement and defining the optimal cut-off point of adequate vitamin D levels are still challenged and intensely debated (21, 24).

It is necessary to establish vitamin references during pregnancy that could provide support for vitamin supplementation programs for pregnant women. In addition, information about the nutritional status of these vitamins during pregnancy based on a large sample survey is lacking in China. Accordingly, the present study aimed to investigate the levels of vitamins A, D, and E in >100,000 pregnant women in western China and to propose reference values of vitamins A, D, and E for women during pregnancy. We also examined the relationship between vitamins and potential influencing factors, including age, pregnancy trimester, season, and regions.

## Materials and Methods

### Population

An epidemiological study was conducted to investigate the levels of three vitamins during pregnancy in women from 17 cities in 4 provinces in western China between 2017 and 2019. Pregnant women who visited the outpatient clinics of the Departments of Obstetrics of Hospitals and underwent serum vitamin measurements were included in the study. The exclusion criteria were (1) missing measurements of vitamin levels, (2) missing information on age, and (3) age < 18 years. The ages, exact time of the vitamin tests, gestational ages, and cities of residence of the pregnant women were collected. The research protocols were approved by the Medical Ethics Committee of the Xi'an Jiaotong University. Written informed consent was obtained from all subjects.

### Laboratory Test

The serum levels of vitamin D2 [25(OH)D2], D3 [25(OH)D3], A (retinol), and vitamin E (α-tocopherol) were assessed using a liquid chromatography mass spectrometer (Shimadzu LC-MS8030, Shimadzu, Kyoto, Japan). Standard curves were established in accordance with the standard material data of the instrument test (*R*^2^ ≥ 0.995). The levels of vitamins D2, D3, A, and E were calculated on the basis of the standard curve function. Vitamin D level was calculated as the total of vitamin D2 and D3 levels.

### Statistical Analyses

Normal distribution was tested with the Kolmogorov–Smirnov test and QQ plot for vitamins A, D, D2, D3, and E. Continuous variables are expressed as means ± standard deviations or medians (interquartile ranges). Categorical variables are presented as numbers (%). The differences in vitamin levels between the multiple groups were compared using analysis of variance for normally distributed data and the Kruskal–Wallis test for non-normally distributed continuous data.

We modeled the trends of the vitamin levels over months of serum collection with restricted cubic spline functions in the linear model. The correlations for age in vitamin A and D levels were fitted with a second-order polynomial. The relationship between age and vitamin E was fitted with a first-order polynomial. Restricted cubic splines with a linear regression model were applied to test the relationship between vitamin levels and other factors, and to determine whether the correlation was linear. Three knots were prespecified at the 5th, 50th, and 95th percentiles of vitamin levels. Multivariable linear models were used to analyze the potential influencing factors of vitamin E level because of the significant linear relationship between age and vitamin E level. Vitamin E was log base 10 transformed before the linear regression analysis because of a right-skewed distribution. Multivariable linear regression models with restricted cubic splines were used to analyze the relationships between vitamin D and A levels because of an evident nonlinear trend between age and vitamin levels. Both models included age, season, gestational trimesters, and regions.

The vitamin reference ranges were defined as vitamin levels within the 2.5th and 97.5th percentiles in the study population and in different seasons and gestational trimesters. Two-sided *p* < 0.05 were considered statistically significant. R 3.5.1, and SPSS 24.0 (SPSS, IL, USA) were used for the statistical analyses in the study.

## Results

### Basic Characteristics of the Study Population

The basic characteristics of the pregnant women are summarized in Table 1. A total of 119,286 pregnant women were included in the study. The mean age of the pregnant women was 28.3 ± 4.5 years. Of these, 107,627 (90.2%) were aged < 35 years and 11,659 (9.8%) were aged ≥ 35 years. Regarding pregnancy stages, 22,800 patients were in their first trimester, 6,999 were in their second trimester, and 14,416 were in their third trimester, accounting for 51.6, 15.8, and 32.6% of the total number of pregnant women in the first, second, and third trimesters, respectively, for whom gestational trimester information was available. In this study, information regarding the exact region was available for 113,523 pregnant women. Most of the pregnant women were from the Shaanxi Province (*n* = 82,735, 72.9%). The number of vitamin measurements in spring (*n* = 30,399, 25.5%), summer (*n* = 33,944, 28.5%), autumn (*n* = 31,403, 26.3%), and winter (23,540, 19.7%) were comparable. The mean levels (mg/L) of vitamins A, E, D, D2, and D3 were 0.4 (0.33–0.46), 12.8 (10.5–15.7), 15.5 (10.5–22.9), 0.6 (0.4–0.9), and 13.4 (9.0–20.0), respectively.

**Table 1:** Basic characteristics of study participants.

**Characteristic**	**Total**	**Year 2017**	**Year 2018**	**Year 2019**
	***n* = 119,286**	***n* = 100,521**	***n* = 8,829**	***n* = 9,936**
**Age (year)**	28.3 ± 4.4	28.3 ± 4.5	28 ± 4.5	28.2 ± 4.2
**Age group**, ***n*****(%)**				
<35	107,627 (90.2%)	90,345 (89.9%)	8,076 (91.5%)	9,206 (92.7%)
≥35	11,659 (9.8%)	10,176 (10.1%)	753 (8.5%)	730 (7.3%)
**Age category**, ***n*****(%)**				
<20	1,230 (1.0)	1,000 (1.0)	139 (1.6)	91 (0.9)
20–	76,625 (64.2)	64,531 (64.2)	5,744 (65.0)	6,350 (63.9)
30–	39,711 (33.3)	33,477 (33.3)	2,823 (32)	3,411 (34.3)
40–	1,720 (1.5)	1,513 (1.5)	123 (1.4)	84 (0.9)
**Gestational age**, ***n*****(%)^a^**				
1st trimester	22,800 (51.6)	19,101 (50.4)	1,715 (48.3)	1,984 (72.3)
2nd trimester	6,999 (15.8)	5,675 (15)	836 (23.6)	488 (17.8)
3rd trimester	14,416 (32.6)	13,145 (34.6)	999 (28.1)	272 (9.9)
**Region**, ***n*****(%)^b^**				
Shaanxi	82,735 (72.9)	66,237 (69.9)	7,715 (87.4)	8,783 (88.4)
Ningxia	5,509 (4.9)	5,154 (5.4)	254 (2.9)	101 (1.0)
Qinghai	10,846 (9.5)	10,139 (10.7)	567 (6.4)	140 (1.4)
Shanxi	14,433 (12.7)	13,228 (14.0)	293 (3.3)	912 (9.2)
**Season of vitamin test**, ***n*****(%)**				
Spring	30,399 (25.5)	24,984 (24.9)	2,680 (30.4)	2,735 (27.5)
Summer	33,944 (28.5)	29,141 (29.0)	2,128 (24.1)	2,675 (26.9)
Autumn	31,403 (26.3)	26,850 (26.7)	2,140 (24.2)	2,413 (24.3)
Winter	23,540 (19.7)	19,546 (19.4)	1,881 (21.3)	2,113 (21.3)
**Vitamin level**				
Vitamin A (mg/L)	0.4 (0.33–0.46)	0.4 (0.33–0.47)	0.38 (0.32–0.44)	0.4 (0.34–0.46)
Vitamin E (mg/L)	12.8 (10.5–15.7)	12.9 (10.6–15.9)	12.1 (10.2–14.8)	11.9 (10–14.4)
Vitamin D (ng/mL)	15.5 (10.5–22.9)	17.8 (11.9–26.7)	14.4 (9.8–20.9)	14.4 (9.9–21)
Vitamin D2 (ng/mL)	0.6 (0.4–0.9)	–	0.6 (0.4–0.9)	0.5 (0.4–0.8)
Vitamin D3 (ng/mL)	13.4 (9–20)	–	13.3 (8.9–20)	13.5 (9–20)

*Age was presented as mean ± standard deviation and vitamin levels were presented as median [interquartile range (IQR)]*.

a 44,215 cases of gestational age information;

b *113,523 cases of region information*.

### Frequency Distribution of the Vitamin Levels

**Figure 1:**
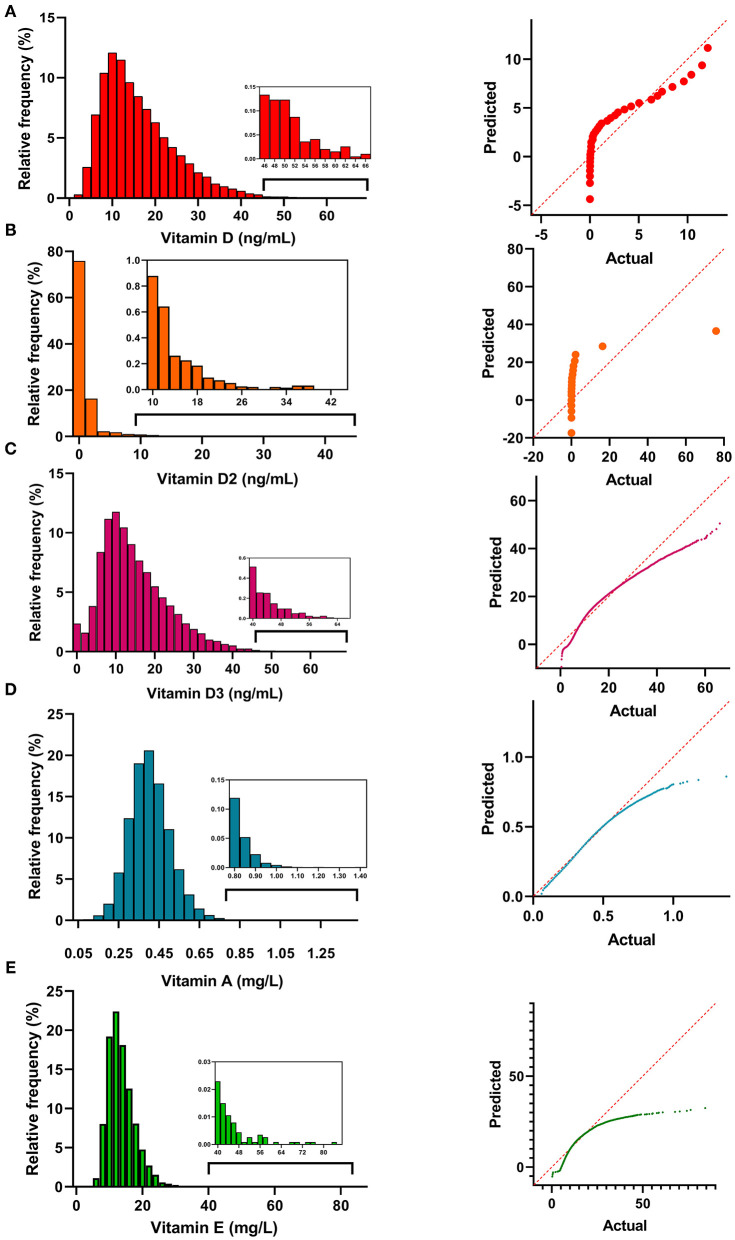
The distribution histograms of vitamin A **(A)**, D2 **(B)**, D3 **(C)**, A **(D)**, and E **(E)**.

The distribution histograms and QQ plots of the vitamin levels are shown in Figure 1. The levels of vitamins A, D, D2, D3, and E showed an abnormal distribution (*p* < 0.001), and were right-skewed. Vitamin A levels had the highest kurtosis (2,314.9 ± 0.01) and skewness values (20.2 ± 0.01), and the vitamin D levels were closer to having a normal distribution (kurtosis: 1.53 ± 0.04; skewness: 1.12 ± 0.02).

### Monthly Variation of the Vitamin Levels

**Figure 2:**
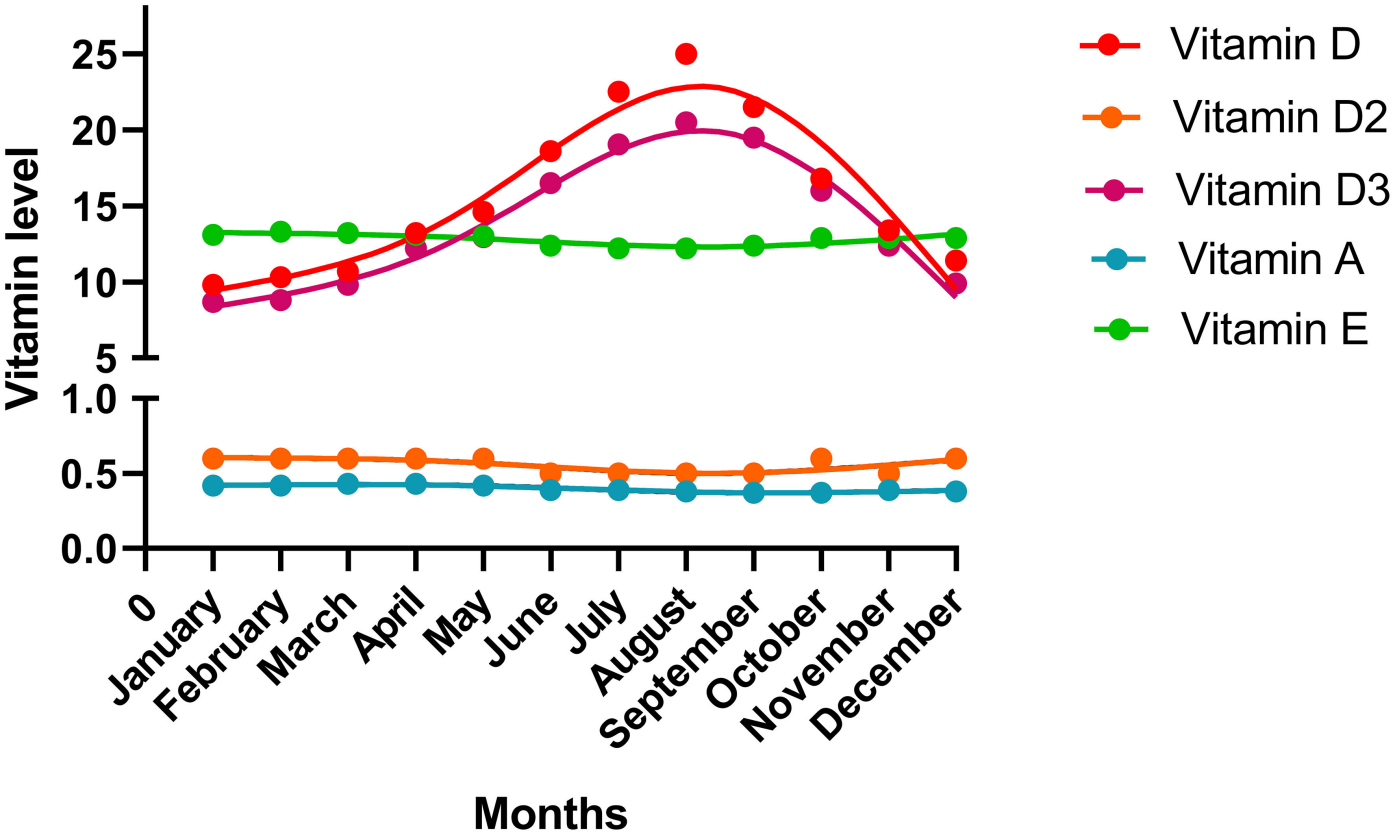
The vitamin levels in the different months. The median value of each month was shown in the plot.

The vitamin levels in different months are shown in Figure 2. Fluctuations in the vitamin D and D3 levels over time are evident. Vitamin D and D3 levels were the lowest in January but increased thereafter. They reached the peak point in August and then declined. The levels of other vitamins showed a trend to remain steady over time.

### Differences in Vitamin Levels Among the Gestational Trimesters

**Figure 3:**
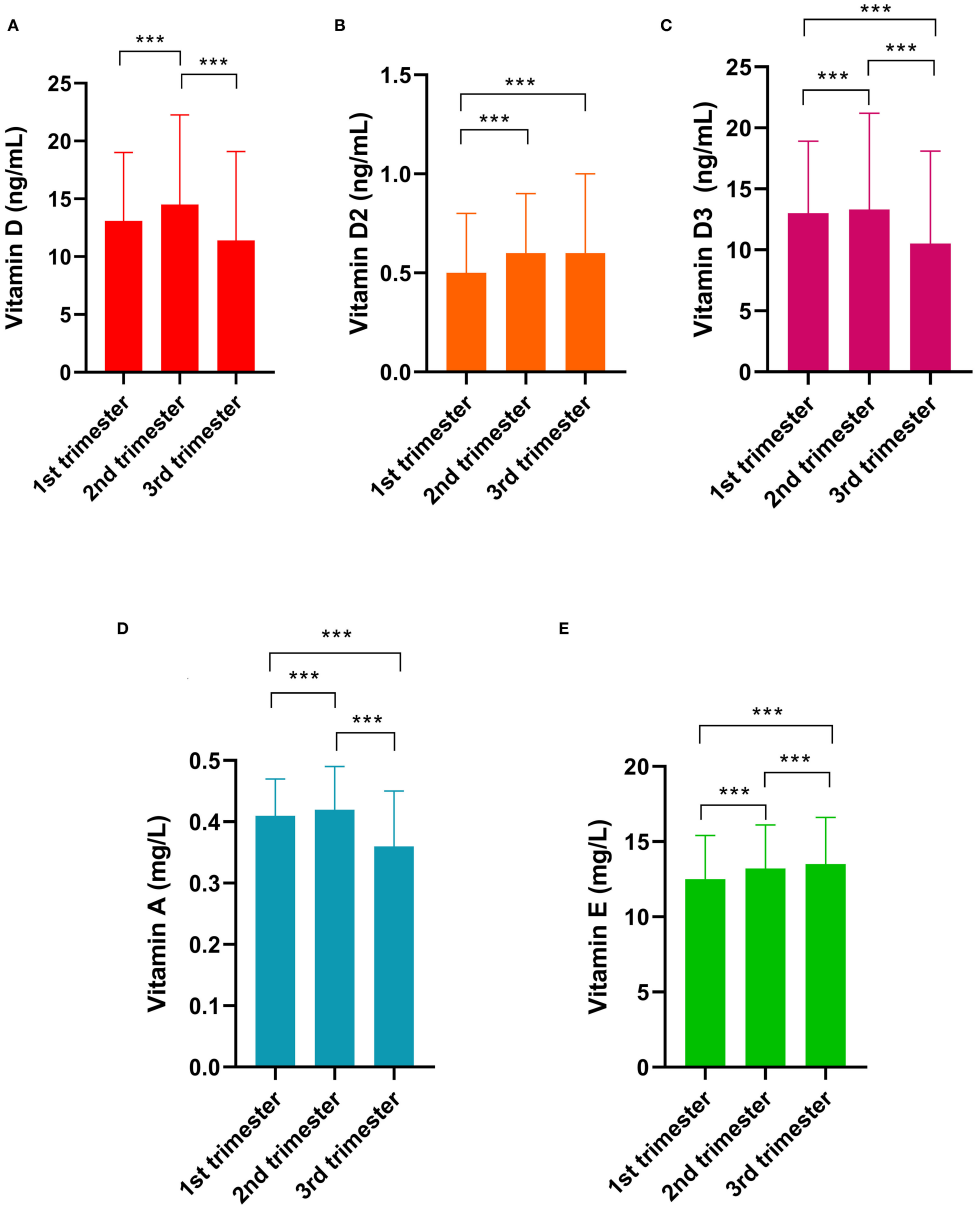
Differences in vitamin levels among the gestational trimesters. **(A)** Vitamin D; **(B)** Vitamin D2; **(C)** Vitamin D3; **(D)** Vitamin A; **(E)** Vitamin E. *** indicates *p* < 0.001.

The differences in vitamin levels among the different trimesters are shown in Figure 3. Vitamin D levels were the highest (14.5 ng/mL) in the second trimester, followed by those in the first (13.1 ng/mL, *p* < 0.001) and third trimesters (11.4 ng/mL, *p* < 0.001). The vitamin D2 level in the first trimester was lower than that in the second and third trimesters (both *p* < 0.001). Vitamin D3 levels were the highest in the second trimester. Vitamin A levels were the highest in the second trimester (0.42 mg/L), followed by those in the first (0.41 mg/L, *p* < 0.001) and third trimesters (0.37 mg/L, *p* < 0.001). The vitamin E levels in the first trimester (11.3 mg/L) was lower than those in the second (13.8 mg/L, *p* < 0.01) and third trimesters (16.0 mg/L, *p* < 0.001).

### Differences in Vitamin Levels Among the Regions

**Figure 4:**
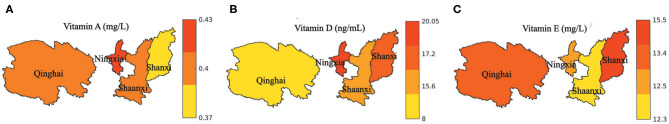
Vitamin levels indifferent regions. **(A)** Vitamin A; **(B)** Vitamin D; **(C)** Vitamin E. The median value of each region was shown in the plot.

The vitamin A, D, and E levels in the different provinces are shown in Figure 4. The vitamin D level was the highest in the Ningxia province (median: 20.05 ng/mL) and the lowest in the Qinghai province (median: 8.0 ng/mL). The vitamin A level was the highest in the Ningxia province (median: 0.43 mg/L) and the lowest in the Shanxi province (median: 0.37 mg/L). The Shanxi province had the highest (median: 15.5 mg/L), while the Shaanxi province had the lowest vitamin E levels (median: 12.3 mg/L). The vitamin D2 and D3 levels in the provinces are shown in Supplementary Figure 1.

**Figure 5:**
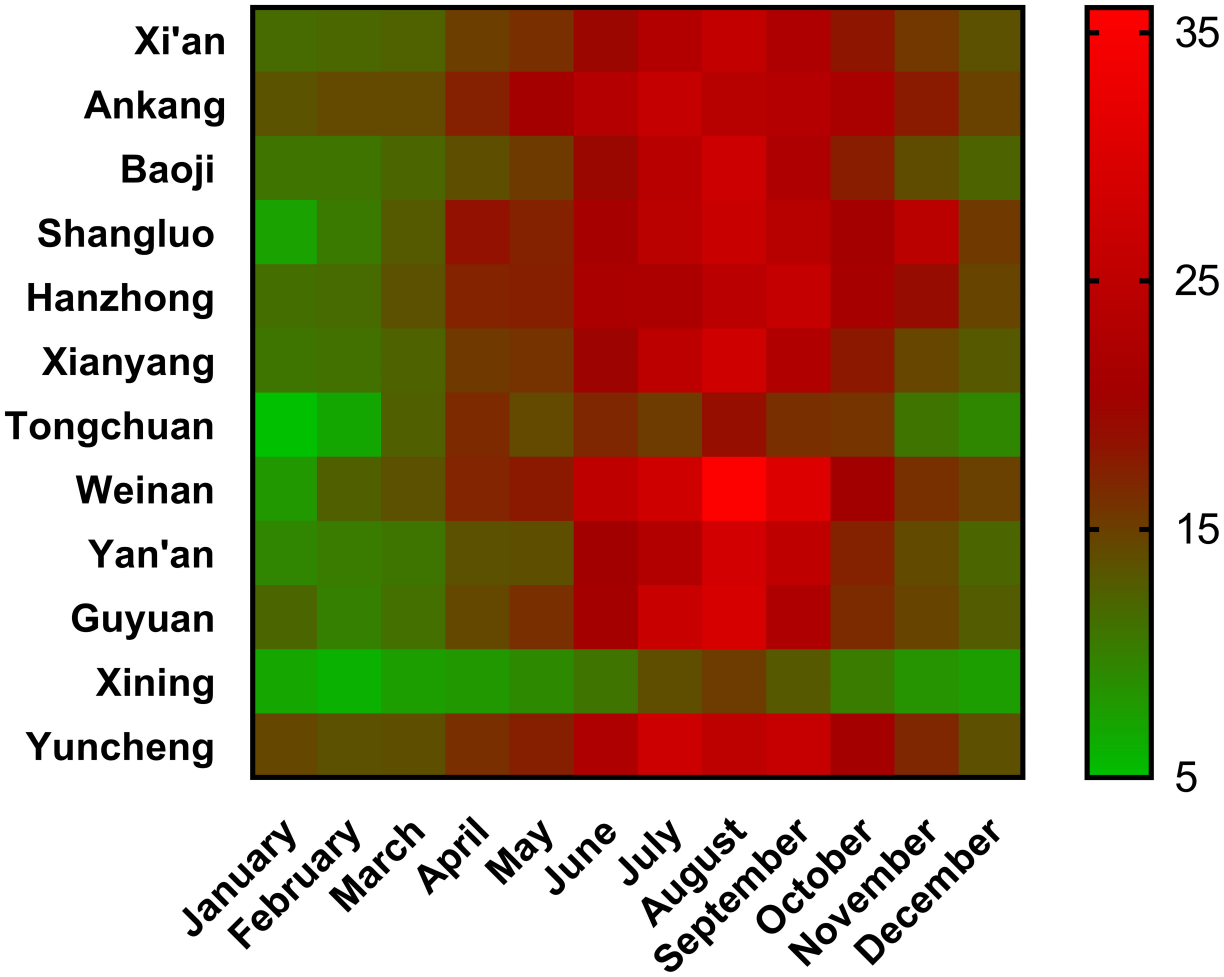
Vitamin D levels in the different cities and months. Median value was shown in each cell.

Vitamin D levels in the different cities and months are shown in Figure 5. Vitamin D levels in Xining were the lowest in all the months. Weinan had the highest vitamin D levels among all the cities. Among 12 cities, 2 cities in July, 9 cities in August, and 1 city in September reached the highest vitamin D level. The vitamin A, D, and E levels in 17 cities are listed in Supplementary Table 1.

### Changes in Vitamin Levels With Age

**Figure 6:**
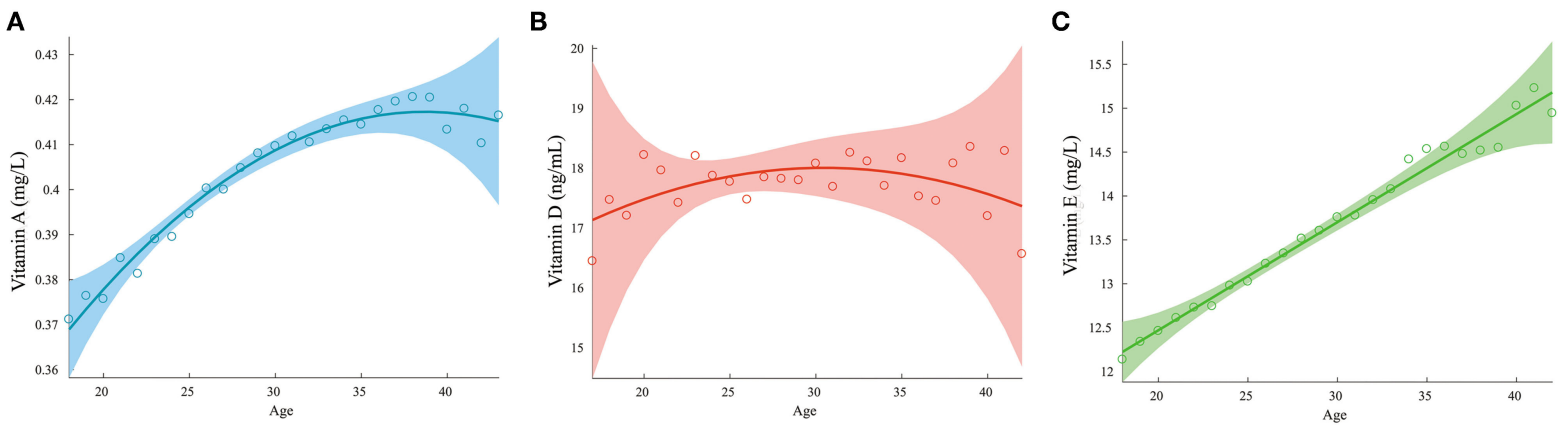
Variation of vitamin levels with age. **(A)** Vitamin A; **(B)** Vitamin D; **(C)** Vitamin E.

The changes in the levels of vitamins A, D, and E with age are shown in Figure 6. The relationships of the vitamin D2 and D3 levels with age are shown in Supplementary Figure 2. The vitamin A and E concentrations increased with age, while pregnant women aged 25–35 years appeared to have higher vitamin D levels than the younger or older pregnant women.

### Multiple Factors Influencing Vitamin Levels

The mean vitamin A, D, and E levels in the different seasons, regions, and gestational trimesters are listed in Supplementary Table 2. The relationships between the potential influencing factors and vitamin E levels (log transformation) are listed in Table 2. The relationships of the potential affecting factors with the vitamin D and A levels with restricted cubic spline function are presented in Table 3. The estimate for age (nonlinear) was statistically significant (*p* < 0.05), which means that the relationship between age and vitamin D and A levels was significantly nonlinear. Age, season, gestational trimesters, and regions were related to vitamin levels.

**Table 2:** Related factors with vitamin E levels with multivariable linear regression model.

**Factors**	**β**	**95%CI**	***p***
**Age**	0.004	0.0037 to 0.0042	<0.001
**Season**			
Spring	−0.002	−0.006 to 0.0008	0.146
Summer	−0.028	−0.031 to −0.024	<0.001
Autumn	−0.013	−0.017 to −0.010	<0.001
Winter	ref		
**Gestational trimester**			
1st trimester	ref		
2nd trimester	0.088	0.085–0.091	<0.001
3rd trimester	0.154	0.151–0.156	<0.001
**Region**			
Shaanxi	−0.030	−0.035 to −0.026	<0.001
Ningxia	−0.063	−0.069 to −0.057	<0.001
Qinghai	−0.027	−0.032 to −0.022	<0.001
Shanxi	ref		

*The four seasons are defined by date: spring, from March to May; summer, from June to August; autumn, from September to November; winter, from December to February*.

**Table 3:** Related factors of vitamin D and vitamin A with multivariable linear regression models and restricted cubic spline.

**Variables**	**Vitamin D**			**Vitamin A**		
	**β**	**95%CI**	***p***	**β**	**95%CI**	***p***
**Age (overall)^a^**	0.16	0.07–0.26	<0.001	0.004	0.004–0.005	<0.001
**Age (non**–**linear)^b^**			0.033			<0.001
**Season**						
Spring	1.99	1.49–2.50	<0.001	0.03	0.02–0.03	<0.001
Summer	9.73	9.21–10.25	<0.001	−0.01	−0.02 to −0.01	<0.001
Autumn	5.78	5.24–6.31	<0.001	−0.02	−0.03 to −0.02	<0.001
Winter	ref			ref		
**Gestational trimester**						
1st trimester	ref			ref		
2nd trimester	3.35	2.93–3.77	<0.001	−0.0003	−0.004 to 0.003	0.864
3rd trimester	5.53	5.08–5.99	<0.001	−0.051	−0.054 to −0.048	<0.001
**Region**						
Shaanxi	10.7	10.1–11.35	<0.001	0.001	−0.005 to 0.006	0.843
Ningxia	–			0.03	0.02–0.04	<0.001
Qinghai	ref			0.02	0.01–0.02	<0.001
Shanxi	–			ref		

*The four seasons are defined by date: spring, from March to May; summer, from June to August; autumn, from September to November; winter, from December to February*.

a overall association;

b *non-linear association*.

### Reference Ranges of the Vitamins

The reference ranges and pregnancy trimester- and season-specific reference ranges of the vitamin levels are listed in Table 4. For vitamin D level, the lower and upper limits were 5 and 43 ng/mL, respectively. The reference ranges for the vitamin A and E levels were 0.22–0.62 and 7.4–23.5 mg/L, respectively. The reference intervals of vitamins in pregnant women without missing values are listed in Supplementary Table 3.

**Table 4:** Reference intervals of vitamin levels in general population and pregnant women.

	**Vitamin D (ng/mL)**	**Vitamin A (mg/L)**	**Vitamin E (mg/L)**
**Reference values in general population**	>15^a^; >25^b^	>0.22^c^	5–18^d^
**Reference values during pregnancy**			
Total population	5–43	0.22–0.62	7.4–23.5
Gestational age			
1st trimester	5.3–35.6	0.26–0.63	7–19.4
2nd trimester	5.3–45	0.24–0.65	8.5–22.5
3rd trimester	3.2–46.8	0.19–0.62	9.4–26.4
Season			
Spring	4.6–33.6	0.23–0.67	7.6–23.9
Summer	7.5–50.4	0.22–0.6	7.2–22.8
Autumn	5.8–41	0.21–0.58	7.4–23.2
Winter	4–31.1	0.22–0.63	7.7–24.2

a *Graham (26)*.

b *Holick et al. (27)*.

c *Bastos Maia et al. (23)*.

d *Kratz et al. (30)*.

## Discussion

The present study summarized the characteristics of vitamins A, D, and E during pregnancy in women from western China and established reference ranges for vitamins A, D, and E during pregnancy based on a large sample size of pregnant women. We also found that the vitamin levels differed according to age, season, gestational trimester, and region.

So far, vitamin deficiency cutoff points for the general population have been defined on the basis of the different nutritional statuses and recommendations by health organizations (13, 14, 23, 27, 28). Pregnancy-specific reference values have not been proposed by official organizations. Researchers suggest that the threshold for vitamin deficiency should be specific in population subgroups, and only one cutoff point for vitamin deficiency across all populations is inappropriate. Vitamin levels may vary according to age, genetics, inflammation, and pregnancy (13). Pregnancy may induce a series of physiological alterations and subsequent changes in biochemical indicators (16). Two recent studies proposed vitamin reference ranges during pregnancy (16, 29). One study enrolled 368 healthy Caucasian pregnant women and proposed gestational trimester-specific reference ranges of vitamin D levels. However, the sample size was not sufficient to establish reference ranges. Another review summarized the reference ranges for all vitamins during pregnancy from different studies. Owing to the different test methods and percentiles defined for the ranges in studies, the reference ranges for vitamin levels from a review with high heterogeneity were difficult to adopt.

Therefore, we proposed the reference ranges of serum vitamin A, D, and E based on a study with a large sample size and a consistent measurement technique to contribute to the management of the micronutritional status of pregnant women in China. In the reference ranges we proposed, the lower limit for vitamin A level was the same as the current vitamin A deficiency cutoff point (0.7 μmol/L; 0.22 mg/L) (23) developed by WHO. The normal value for vitamin E in the general population is 5–18 mg/L (30). The lower limit of vitamin E levels in our study was higher. One study found that high maternal vitamin E supplements were related to an increased risk of congenital heart defects (31). This supports our result that pregnant women maintain higher vitamin E levels than the general population owing to their pregnancy and may not need additional vitamin E supplementation. Moreover, the lower limit of the vitamin D levels in our study (5 ng/mL) was much lower than the current vitamin D deficiency cutoff point (30 nmol/L; 15 ng/mL) developed by the Institute of Medicine (28) or the cutoff point (50 nmol/L; 25 ng/mL) developed by the Endocrine Society (27), which suggests that the current cutoff point for vitamin D deficiency is not suitable for pregnant women. Whether vitamin D supplementation during pregnancy is needed is still a controversial issue (32). The WHO has insufficient evidence to recommend vitamin D supplementation during pregnancy to protect against adverse outcomes (13, 21, 24). This may be related to an inappropriate threshold for vitamin D deficiency.

We found that vitamin levels during pregnancy differed according to age, season, region, and gestational trimesters. Vitamin A levels were lower in the third trimester than in the first and second trimesters, which is in agreement with numerous previous reports (17, 23, 33–35). This may be ascribed to the increased plasma volume and accelerated fetal consumption with increasing gestational age (17, 23). Nevertheless, vitamin D level had a positive correlation with gestational trimester. One possible reason is that most pregnant women in China follow their physician's order to take calcium supplements with vitamin D at the mid and late stages of pregnancy. Recent studies have reported that vitamin D supplementation during pregnancy could increase maternal and infant vitamin D concentrations and improve maternal insulin resistance and fetal growth (24, 36). Although vitamin E supplementation during pregnancy was not recommended, we still found that vitamin E levels increased with gestational trimester. This may be related to its increased absorption efficiency during the course of pregnancy. To maintain pregnancy and antioxidant function, the absorption of vitamin E may increase with gestational age (14, 37). One of the vitamin E transporters vitamin E-binding protein afamin has been reported to increase in maternal blood with gestational age, which could contribute to the absorption of vitamin E (25).

Besides gestational trimester, vitamin D and D3 levels were found to be altered over months and seasons. The reason may be that vitamin D3, which can be synthesized in the skin by exposure to sunlight, is the major component of vitamin D. Vitamin D2 can be of plant origin (14). Therefore, vitamin D3 is affected more by sunlight and season than vitamin D2. In terms of the influence of districts, we found significant differences in vitamin A, D, and E levels among provinces, which may be attributed to the duration of sunlight exposure, clothing, behavior, and maternal health policies in different regions. Regarding age, we found nonlinear relationships between vitamin A and D levels and age, and a positive linear relationship between vitamin E level and age. The reason for the change in regulation of the vitamin levels with age is unclear. Previous studies have reported that increased plasma levels of 1, 25 (OH)_2_ vitamin D3 (calcitriol) and 25(OH)D are related to increased circulating estrogen levels (38–40). Moreover, estrogen levels increase and reach peak values in the mid- to late 20s in women and then decline (41). Accordingly, we speculate that vitamin D levels increased until 30 years of age and decreased thereafter, owing to the change in hormonal status, especially estrogen level.

The present study has some limitations. First, the adverse maternal and fetal outcomes of the study population were not acquired in this study. In fact, the rate of birth defects in China has remained 5.6% since 2012. The present vitamin levels during pregnancy in China did not influence the fluctuation in the rate of birth defects. Numerous studies have already demonstrated that vitamin A, D, and E levels are related to congenital malformation (23, 31, 42, 43). Accordingly, at the macroscopic level, it may indicate that current vitamin A, D, and E levels during pregnancy in China may be safe, and vitamin reference ranges may represent levels of most normal pregnant women. Although the reference ranges we proposed may be not precise, it suggests the need for pregnancy-specific reference ranges for vitamins. Second, although several influencing factors of vitamin concentrations have already been taken into account, some potential confounders remain, such as income, educational level, and vitamin supplementation during pregnancy, which were not included in the study. Finally, we did not include all the provinces of China in this study. The study population was not randomly selected. Further studies with a probability sampling design to investigate vitamin levels during pregnancy are needed.

In conclusion, the reference value for vitamin A during pregnancy was the same as the currently used reference value in the general population. The reference values for vitamins D and E during pregnancy were different from the currently used reference values for the general population, which may indicate the necessity for establishing pregnancy-specific reference ranges for vitamins D and E. Vitamin A, D, and E levels differed according to age, gestational trimester, season, and region. Evidence from our study may contribute to future micronutritional management of pregnant women.

## Data Availability Statement

The raw data supporting the conclusions of this article will be made available by the authors, without undue reservation, to any qualified researcher.

## Ethics Statement

The studies involving human participants were reviewed and approved by the medical ethics committee of the First Affiliated Hospital of Xi'an Jiaotong University. The patients/participants provided their written informed consent to participate in this study.

## Author Contributions

GB and DC worked on the study design, data interpretation, and manuscript review. FGa, FGu, YZ, YY, and GB contributed to obtaining data from the participants recruited in the study. FGa and FGu contributed to the data analysis. FGa wrote the first draft of the manuscript, and all other authors provided additional suggestions. All authors approved the final version of the manuscript.

## Conflict of Interest

The authors declare that the research was conducted in the absence of any commercial or financial relationships that could be construed as a potential conflict of interest.
