# Supplementary material for: Vitamin A, D, and E Levels and Reference Ranges for Pregnant Women: A Cross-Sectional Study 2017–2019
Source: Front Nutr. 2021 Mar 22;8:628902. doi: 10.3389/fnut.2021.628902 (PMC8019719; doi:10.3389/fnut.2021.628902)
Supplement: Supplementary file 1 [file Table_1.DOCX]

| Table S1 Vitamin levels in different cities. | | | | | | | | | |
| --- | --- | --- | --- | --- | --- | --- | --- | --- | --- |
| Province | City | Vitamin D (ng/mL) | |  | Vitamin A (mg/L) | |  | Vitamin E (mg/L) | |
|  |  | Medium | Q1-Q3 |  | Medium | Q1-Q3 |  | Medium | Q1-Q3 |
| Shaanxi | Xi'an | 15.3 | 10.4-22.4 |  | 0.4 | 0.34-0.47 |  | 12.5 | 10.3-15.3 |
|  | Ankang | 16.4 | 11.9-22.6 |  | 0.41 | 0.35-0.48 |  | 12 | 10.2-14.4 |
|  | Baoji | 15.2 | 10.6-22.7 |  | 0.41 | 0.35-0.47 |  | 12.1 | 10.3-14.6 |
|  | Shangluo | 19.2 | 12.4-24.5 |  | 0.38 | 0.32-0.45 |  | 12 | 10.1-14.3 |
|  | Hanzhong | 15.4 | 10.8-21.8 |  | 0.39 | 0.33-0.45 |  | 12.5 | 10.6-14.9 |
|  | Xianyang | 15.8 | 10.5-23.7 |  | 0.39 | 0.33-0.45 |  | 11.8 | 9.8-14.4 |
|  | Tongchuan | 13.6 | 8.4-19.2 |  | 0.37 | 0.33-0.43 |  | 10.3 | 8.8-12.2 |
|  | Weinan | 19.1 | 13.4-28 |  | 0.39 | 0.35-0.46 |  | 11.9 | 10-13.6 |
|  | Yan'an | 14.6 | 9.9-21.8 |  | 0.4 | 0.33-0.47 |  | 12.6 | 10.3-15.5 |
|  | Yulin | 16.9 | 11.8-21.5 |  | 0.41 | 0.37-0.45 |  | 13 | 10.4-15.2 |
|  |  |  |  |  |  |  |  |  |  |
| Ningxia | Guyuan | 16.4 | 10.5-23.9 |  | 0.37 | 0.31-0.44 |  | 12.4 | 9.8-14.7 |
|  | Wuzhong | 23.1 | 15.8-23.6 |  | 0.45 | 0.39-0.52 |  | 11 | 9.5-12.8 |
|  | Yinchuan | 27.6 | 19.5-32.8 |  | 0.42 | 0.34-0.5 |  | 14.1 | 11.7-16.8 |
|  | Zhongwei | 22.9 | 13.7-29.8 |  | 0.44 | 0.38-0.49 |  | 10.3 | 8.8-12.1 |
|  |  |  |  |  |  |  |  |  |  |
| Qinghai | Xining | 8 | 5.2-12.3 |  | 0.4 | 0.33-0.47 |  | 13.5 | 11.1-16.5 |
|  |  |  |  |  |  |  |  |  |  |
| Shanxi | Yuncheng | 17.2 | 12.2-23.6 |  | 0.37 | 0.30-0.44 |  | 15.8 | 12.7-19.3 |
|  | Linfen | 18 | 13.5-22.1 |  | 0.37 | 0.30-0.44 |  | 15.2 | 12.4-18.5 |
