# Supplementary material for: Vitamin A, D, and E Levels and Reference Ranges for Pregnant Women: A Cross-Sectional Study 2017–2019
Source: Front Nutr. 2021 Mar 22;8:628902. doi: 10.3389/fnut.2021.628902 (PMC8019719; doi:10.3389/fnut.2021.628902)
Supplement: Supplementary file 2 [file Table_2.DOCX]

| Table S2 Vitamin A, D and E average levels in different season, region and gestational trimester. | | | | | | | |  |
| --- | --- | --- | --- | --- | --- | --- | --- | --- |
| Case-type | Group1 | Group2 | Vitamin A (mg/L) | | Vitamin D (ng/mL) | | Vitamin E (mg/L) | |
|  |  |  | Medium | Q1-Q3 | Medium | Q1-Q3 | Medium | Q1-Q3 |
| **Season-Region** | Spring | Shaanxi | 0.43 | 0.36-0.5 | 13.1 | 9.6-18.2 | 12.6 | 10.5-15.4 |
|  |  | Ningxia | 0.44 | 0.36-0.52 | 14 | 9-19.4 | 13.9 | 11.4-16.8 |
|  |  | Qinghai | 0.44 | 0.36-0.52 | 7.2 | 4.9-10.8 | 13.6 | 11.3-16.8 |
|  |  | Shanxi | 0.4 | 0.33-0.48 | 14.6 | 11.3-19.5 | 15.9 | 12.8-19.3 |
|  | Summer | Shaanxi | 0.39 | 0.33-0.45 | 21.5 | 15.5-29.3 | 11.9 | 9.9-14.6 |
|  |  | Ningxia | 0.41 | 0.35-0.48 | 28.7 | 21.4-37.5 | 11.3 | 9.4-13.9 |
|  |  | Qinghai | 0.39 | 0.33-0.46 | 11.2 | 7.4-16.9 | 12.8 | 10.5-15.6 |
|  |  | Shanxi | 0.36 | 0.3-0.43 | 22.1 | 17.8-28.3 | 15.1 | 12.2-18.3 |
|  | Autumn | Shaanxi | 0.38 | 0.32-0.44 | 17.2 | 12.2-23.8 | 12.2 | 10.2-14.8 |
|  |  | Ningxia | 0.41 | 0.35-0.47 | 18.9 | 13.6-25.5 | 11.8 | 9.7-14.4 |
|  |  | Qinghai | 0.38 | 0.32-0.45 | 9.3 | 5.9-13.6 | 13.5 | 11.2-16.5 |
|  |  | Shanxi | 0.35 | 0.29-0.42 | 19.1 | 13.9-25.6 | 15.4 | 12.5-18.7 |
|  | Winter | Shaanxi | 0.4 | 0.34-0.47 | 10.5 | 7.7-14.7 | 12.7 | 10.6-15.4 |
|  |  | Ningxia | 0.4 | 0.33-0.47 | 12 | 8-16.6 | 12.7 | 10.4-15 |
|  |  | Qinghai | 0.39 | 0.32-0.46 | 5.7 | 3.6-8.4 | 14.1 | 11.4-17.3 |
|  |  | Shanxi | 0.36 | 0.3-0.44 | 12.5 | 9.2-16.7 | 15.8 | 12.8-19.3 |
| **Season-Pregnancy** | Spring | 1st trimester | 0.44 | 0.37-0.51 | 12.6 | 9.3-17.2 | 11.6 | 9.9-13.7 |
|  |  | 2nd trimester | 0.44 | 0.38-0.52 | 13.5 | 9.8-18.8 | 13.8 | 11.7-16.4 |
|  |  | 3rd trimester | 0.39 | 0.32-0.48 | 11.5 | 7.4-17.1 | 16.3 | 13.6-19.3 |
|  | Summer | 1st trimester | 0.4 | 0.34-0.46 | 19 | 13.9-25.1 | 10.6 | 9.1-12.4 |
|  |  | 2nd trimester | 0.4 | 0.35-0.46 | 23.2 | 16.8-32.6 | 13.8 | 11.8-15.9 |
|  |  | 3rd trimester | 0.35 | 0.29-0.42 | 21.4 | 13-31.8 | 15.9 | 13.6-18.6 |
|  | Autumn | 1st trimester | 0.39 | 0.34-0.45 | 15.3 | 11-20.9 | 11.2 | 9.6-13 |
|  |  | 2nd trimester | 0.39 | 0.34-0.46 | 18.5 | 12.4-25.4 | 13.9 | 12-16.2 |
|  |  | 3rd trimester | 0.34 | 0.28-0.41 | 16.4 | 10.6-25.4 | 16.2 | 14-18.7 |
|  | Winter | 1st trimester | 0.42 | 0.36-0.48 | 9.8 | 7.3-13.5 | 12 | 10.2-14.4 |
|  |  | 2nd trimester | 0.42 | 0.36-0.5 | 11.5 | 7.8-17.2 | 13.9 | 11.7-16.4 |
|  |  | 3rd trimester | 0.39 | 0.31-0.47 | 10.7 | 6.5-16.6 | 15.5 | 12.8-18.5 |
| **Pregnancy-Region** | 1st trimester | Shaanxi | 0.4 | 0.35-0.47 | 14.4 | 10-20.4 | 11.2 | 9.5-13.1 |
|  |  | Ningxia | 0.41 | 0.35-0.48 | - |  | 9.3 | 7.9-11.2 |
|  |  | Qinghai | 0.43 | 0.37-0.49 | 8.5 | 6-12.2 | 11.4 | 9.8-13.3 |
|  |  | Shanxi | 0.4 | 0.35-0.46 | - |  | 11.8 | 10.2-13.7 |
|  | 2nd trimester | Shaanxi | 0.41 | 0.35-0.48 | 16.8 | 11-24.6 | 13.5 | 11.4-15.9 |
|  |  | Ningxia | 0.45 | 0.39-0.53 |  |  | 14 | 12.2-16.1 |
|  |  | Qinghai | 0.42 | 0.35-0.49 | 9.1 | 6.5-13.8 | 14.2 | 12.3-16.7 |
|  |  | Shanxi | 0.4 | 0.34-0.47 |  |  | 14.7 | 12.8-17.2 |
|  | 3rd trimester | Shaanxi | 0.36 | 0.3-0.43 | 18.7 | 12.6-27.8 | 16.5 | 14.1-19.2 |
|  |  | Ningxia | 0.39 | 0.31-0.48 |  |  | 15.1 | 12.7-17.7 |
|  |  | Qinghai | 0.36 | 0.29-0.44 | 7.9 | 5-12.3 | 15.8 | 13.4-18.8 |
|  |  | Shanxi | 0.34 | 0.29-0.41 |  |  | 16.9 | 14.6-19.8 |
