# Supplementary material for: Vitamin A, D, and E Levels and Reference Ranges for Pregnant Women: A Cross-Sectional Study 2017–2019
Source: Front Nutr. 2021 Mar 22;8:628902. doi: 10.3389/fnut.2021.628902 (PMC8019719; doi:10.3389/fnut.2021.628902)
Supplement: Supplementary file 3 [file Table_3.DOCX]

| Table S3 Reference intervals of vitamin levels in general population and pregnant women without missing value. | | | |
| --- | --- | --- | --- |
|  | Vitamin D  (ng/mL) | Vitamin A  (mg/L) | Vitamin E  (mg/L) |
|  |  |  |  |
| **Reference values in general population** | >15^a^; >25 ^b^ | >0.22 ^c^ | 5-18 ^d^ |
| **Reference values during pregnancy** |  |  |  |
| Total population | 4.4-42.2 | 0.22-0.63 | 7.5-23.3 |
| Gestational age |  |  |  |
| 1st trimester | 5.3-35.6 | 0.26-0.63 | 7-19.1 |
| 2nd trimester | 5.3-45.1 | 0.24-0.64 | 8.6-22.4 |
| 3rd trimester | 3.2-47 | 0.19-0.61 | 9.6-26.4 |
| Season |  |  |  |
| Spring | 4.5-34.9 | 0.24-0.67 | 7.6-23.9 |
| Summer | 5.4-41.8 | 0.22-0.59 | 7.2-22.6 |
| Autumn | 5.2-40.4 | 0.21-0.58 | 7.5-23.0 |
| Winter | 3.3-32.8 | 0.22-0.63 | 7.7-24.1 |
| a. Graham L. IOM Releases Report on Dietary Reference Intakes for Calcium and Vitamin D. American Family Physician. 2011;83(11):1352-1352. b. Holick MF, Binkley NC, Bischoff-Ferrari HA, Gordon CM, Hanley DA, Heaney RP, Murad MH, Weaver CM, Endocrine S. Evaluation, treatment, and prevention of vitamin D deficiency: an Endocrine Society clinical practice guideline. J Clin Endocrinol Metab. 2011;96(7):1911-1930. c. Bastos Maia S, Rolland Souza AS, Costa Caminha MF, Lins da Silva S, Callou Cruz R, Carvalho Dos Santos C, Batista Filho M. Vitamin A and Pregnancy: A Narrative Review. Nutrients. 2019;11(3). d. Kratz A, Ferraro M, Sluss PM, Lewandrowski KB. Laboratory reference value. New England Journal of Medicine. 2004;351(15):1548-1563. | | | |
