# Supplementary figures and images for: Vitamin A, D, and E Levels and Reference Ranges for Pregnant Women: A Cross-Sectional Study 2017–2019
Source: Front Nutr. 2021 Mar 22;8:628902. doi: 10.3389/fnut.2021.628902 (PMC8019719; doi:10.3389/fnut.2021.628902)

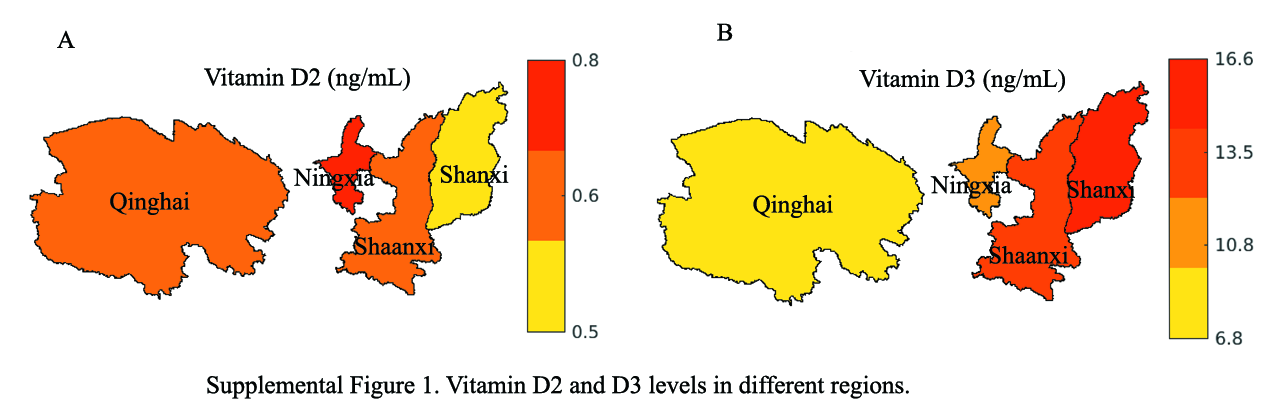

Supplement: Supplementary file 4 [file Image_1.TIF]

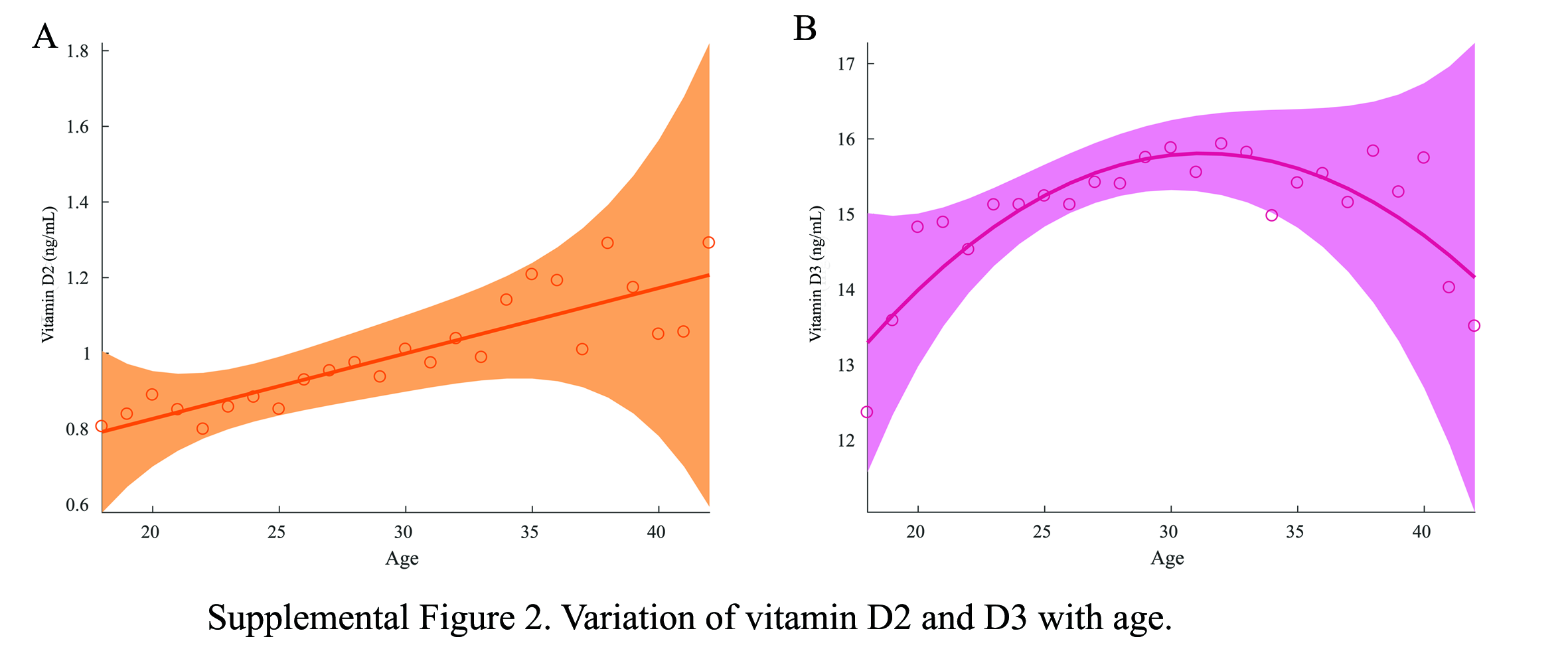

Supplement: Supplementary file 5 [file Image_2.TIF]
